# Reduced Sialylation of Airway Mucin Impairs Mucus Transport by Altering the Biophysical Properties of Mucin

**DOI:** 10.21203/rs.3.rs-4421613/v1

**Published:** 2024-05-31

**Authors:** Elex S. Harris, Hannah J. McIntire, Marina Mazur, Hinnerk Schulz-Hildebrandt, Hui Min Leung, Guillermo J Tearney, Stefanie Krick, Steven M. Rowe, Jarrod W. Barnes

**Affiliations:** Gregory Fleming James Cystic Fibrosis Research Center, Univ. of Alabama at Birmingham, Birmingham, AL, USA; Gregory Fleming James Cystic Fibrosis Research Center, Univ. of Alabama at Birmingham, Birmingham, AL, USA; Gregory Fleming James Cystic Fibrosis Research Center, Univ. of Alabama at Birmingham, Birmingham, AL, USA; Massachusetts General Hospital, Boston, MA, USA.; Massachusetts General Hospital, Boston, MA, USA.; Massachusetts General Hospital, Boston, MA, USA.; Gregory Fleming James Cystic Fibrosis Research Center, Univ. of Alabama at Birmingham, Birmingham, AL, USA; Gregory Fleming James Cystic Fibrosis Research Center, Univ. of Alabama at Birmingham, Birmingham, AL, USA; Gregory Fleming James Cystic Fibrosis Research Center, Univ. of Alabama at Birmingham, Birmingham, AL, USA

## Abstract

Mucus stasis is a pathologic hallmark of muco-obstructive diseases, including cystic fibrosis (CF). Mucins, the principal component of mucus, are extensively modified with hydroxyl (O)-linked glycans, which are largely terminated by sialic acid. Sialic acid is a negatively charged monosaccharide and contributes to the biochemical/biophysical properties of mucins. Reports suggest that mucin sialylation may be altered in CF; however, the consequences of reduced sialylation on mucus clearance have not been fully determined. Here, we investigated the consequences of reduced sialylation on the charge state and conformation of the most prominent airway mucin, MUC5B, and defined the functional consequences of reduced sialylation on mucociliary transport (MCT). Reduced sialylation contributed to a lower charged MUC5B form and decreased polymer expansion. The inhibition of total mucin sialylation *de novo* impaired MCT in primary human bronchial epithelial cells and rat airways, and specific α−2,3 sialylation blockade was sufficient to recapitulate these findings. Finally, we show that ST3 beta-galactoside alpha-2,3-sialyltransferase (ST3Gal1) expression is downregulated in CF and partially restored by correcting CFTR via Elexacaftor/Tezacaftor/Ivacaftor treatment. Overall, this study demonstrates the importance of mucin sialylation in mucus clearance and identifies decreased sialylation by ST3Gal1 as a possible therapeutic target in CF and potentially other muco-obstructive diseases.

## Introduction

Cystic fibrosis (CF) is a genetic disease hallmarked by viscous and adhesive airway mucus in several organs (1–3). In the respiratory tract, CF mucus becomes static and leads to chronic infection, progressive organ decline, and early mortality (1, 4). The rheological characteristics of the mucus gel are largely governed by the gel-forming mucins MUC5B and MUC5AC, the chief structural component of mucus (2, 5). In CF, electrostatic driven abnormalities, stemming from impaired anion transport, alter mucin conformation and contribute to increased mucus viscosity and impaired mucociliary clearance (4, 6–9).

Gel-forming mucins are extensively modified with hydroxyl (O)-linked glycans that determine biophysical properties including normal mucin expansion and rheological characteristics (5, 10, 11). Mucin glycans are largely terminated by sialic acid attached in either an α−2,3 or α−2,6 linkage, facilitated by either ST3 beta-galactoside alpha-2,3-sialyltransferase (ST3Gal) or ST6 N-acetylgalactosaminide alpha-2,6-sialyltransferase (ST6GalNAc), respectively (12, 13). Sialic acid is a negatively charged monosaccharide and exerts key electrostatic properties to mucins via its negative charge (14). The high anionic density of mucins is postulated to help stiffen the mucin polymer through charge repulsion and mediate interactions with cations to facilitate mucin granular packaging and post-secretory expansion (10, 15–17). Within intracellular granules, calcium (Ca^2+^) shields these negative charges to mitigate electrostatic repulsion and promote mucin condensation (11, 18). Upon secretion, divalent sodium (Na^+^) is exchanged with Ca^2+^ to facilitate mucin expansion and hydration (5, 6). Although anionic density is central to the mechanisms of mucin maturation and hydration, studies have historically centered around the altered ionic environment as the driver of aberrant mucin in CF, and the role of intrinsic mucin sialylation/charge in mucin biogenesis and MCC has remained understudied.

Previous work has implicated lower charged mucin in several muco-obstructive diseases. The predominant gel-forming mucin of the airway, MUC5B, has been characterized to exist in two forms: (1) a more negative “high” charged form, and (2) a less negative “low” charged form (based on its migration following Agarose-PAGE) (19). Increased levels of the low charged form have been reported in CF, asthma, and COPD (5, 20, 21). In asthmatics, the low charged form was enriched within a viscous mucus plug and exhibited a compact and entangled conformation, linking decreased MUC5B charge with conformational and rheological defects (22). However, the etiology of this MUC5B form and the functional consequences of reduced/low mucin charge on polymer expansion and MCC have not been elucidated. Interestingly, changes in mucin sialylation have been documented in CF. Specifically, evidence supports that sialylation may be reduced as a direct consequence of defective CFTR, although the role of CFTR in regulating mucin sialylation/charge and its impact on CF mucus pathology remain unclear (23–26).

In this study, we aimed to determine the role of mucin sialylation on the biophysical properties of mucin and overall mucus function in terms of charge state, compaction, and mucus transport. Furthermore, we aimed to determine the clinical relevance of aberrant mucin sialylation in CF. Here, we show that reduced sialylation of mucin contributed to a low charged form of MUC5B, increased MUC5B compaction, and ultimately impaired mucociliary transport *in vitro* and *in vivo*. Additionally, we provide evidence for a link between the defective CFTR and reduced sialylation of mucin in CF mucus stasis. Overall, this study demonstrates the importance of sialylation in mucus function and provides impetus to investigate the molecular mechanisms of mucin sialylation for treatment of mucociliary dysfunction in CF, as well as potentially other muco-obstructive diseases.

## Results

### Reducing the Sialylation of Contributes to a Low Charge Form of MUC5B

**Figure 1:**
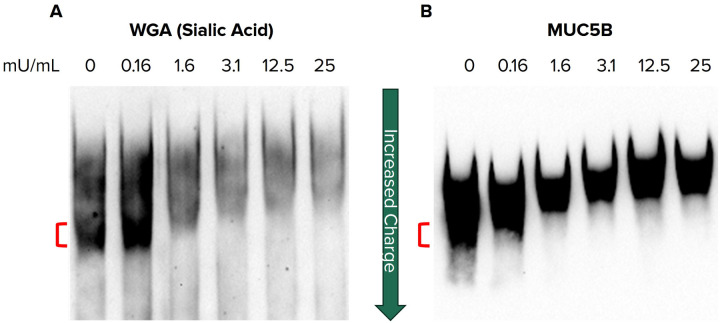
Reducing the sialylation of secreted mucin contributes to a low charge form of MUC5B Agarose-PAGE western blots of partially purified mucin from non-CF HBEC secretions. Mucin was treated with increasing concentrations of neuraminidase, ranging from 0 to 25mu/mL, to remove sialic acid and separated by gel electrophoresis before being probed for **(A)** sialic acid (WGA) and **(B)**MUC5B. The faster migrating/highest charged species (red bar) disappears as sialic acid is increasingly removed. The gel mobility of MUC5B is decreased as sialic acid is removed indicating a decrease in charge.

Since sialic acid contributes to the overall charge of MUC5B, we hypothesized that reducing sialylation would result in a lower charged mucin, similar to the predominate forms in COPD and asthma (5, 22, 27). To test this, we collected and partially purified mucin from non-CF HBEC secretions, then treated this mucin with increasing concentrations of sialidase. Using a agarose polyacrylamide gel electrophoresis gel mobility assay to characterize mucin charge state (28), sialidase treated mucins were separated followed by sialylation analysis with WGA lectin blotting (Fig. 1A). As sialylation was reduced by sialidase treatment, the faster migrating mucin forms were dose-dependently shifted to a slower mobility in the gel (Fig. 1A) indicative of charge reduction. In parallel experiments, an incremental shift in gel mobility of MUC5B was observed, reflecting the dose-dependent loss of sialic acid following sialidase treatment (Fig. 1B). Together, these results provide evidence that sialylation is important in the charge state of mucins and its reduction may contribute to the low charged MUC5B form previously implicated in muco-obstructive disease.

### Reducing Sialylation of Salivary MUC5B Impairs Mucin Linearization

**Figure 2:**
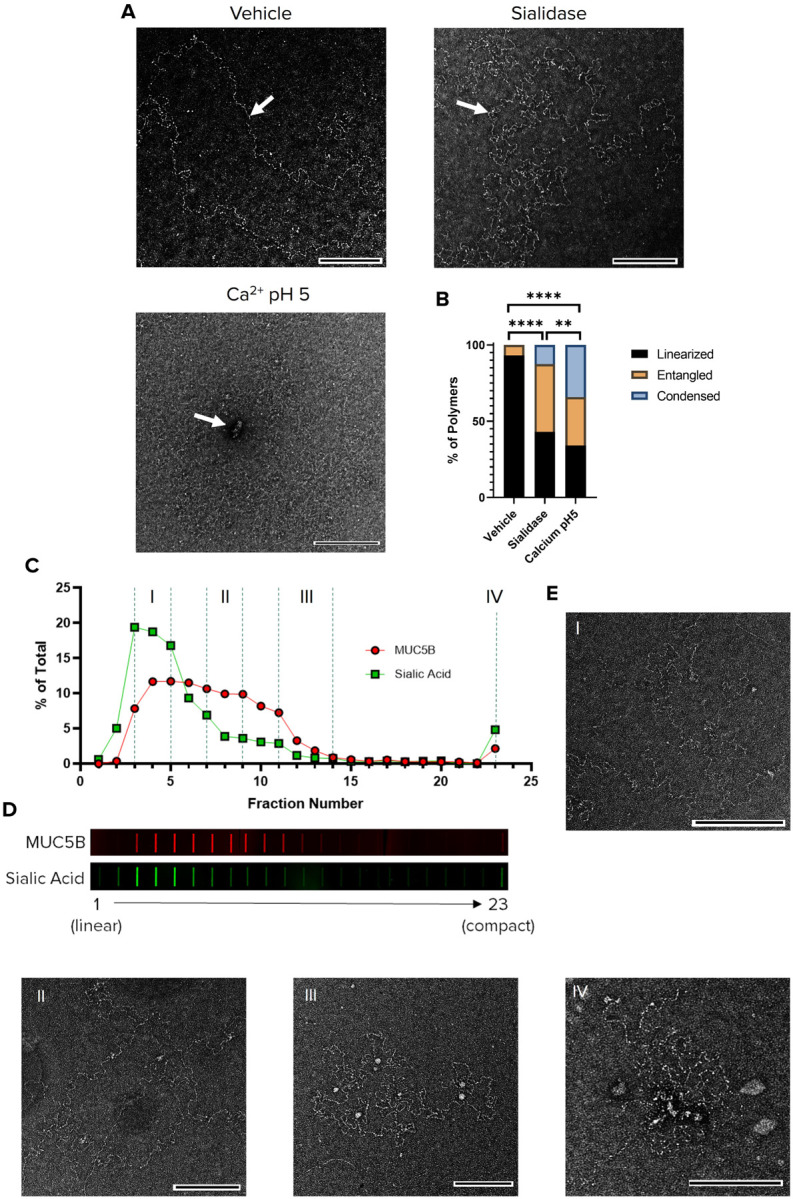
Reducing Sialylation of MUC5B Impairs Mucin Linearization **(A-B)** Natively purified MUC5B was treated with either vehicle or sialidase at pH 7.4 or 10mM CaCl_2_ at a pH of 5. 10mM EGTA at pH 7.4 was added to vehicle and sialidase groups prior to ON incubation at 4°C. MUC5B polymers were subsequently visualized by negative stain TEM. **(A)** representative TEM images of MUC5B polymers treated with either vehicle, sialidase, or calcium CaCl_2_/pH 5. Imaging was performed to capture 72–85 polymers per group (vehicle, N=73; sialidase, N=72; and 10mM CaCl_2_/pH 5, N=85). After blinding, polymers were counted and categorized into 3 groups for each condition: linear, entangled, or condensed. White arrows indicate a linearized polymer in the vehicle group, an entangled polymer in the sialidase group, and a condensed polymer in the CaCl_2_/pH 5 group. **(B)** Quantification showing the percentage total polymers that were linear, entangled, or condensed for each group. N=72–85 per group. **P<0.01, ****P<0.0001 by Chi Square. Scale bars, 200 nm. **(C-D)** Untreated MUC5B was separated on 10–35% Sucrose gradient by rate zonal centrifugation to separate MUC5B by shape; more compact mucins sediment faster. Gradient fractions were slot blotted and probed for MUC5B and sialic acid content. **(C)** Intensities were quantified as a percentage of whole for both MUC5B and sialic acid. **(D)** Representative slot blots of MUC5B and sialic acid, showing a higher percentage of sialic acid content compared to MUC5B in the early (more linear) fractions. (E) Fractions were pooled to represent 4 different sedimentation rates across the gradient and imaged via TEM. Scale bars, 200 nm.

To determine the consequences of reduced sialylation on MUC5B conformation, we natively purified salivary MUC5B via CsCl gradient centrifugation as previously described, incubated it with either sialidase or vehicle, and subsequently evaluated the macromolecular conformations of MUC5B polymers via TEM (29). In parallel, we incubated untreated MUC5B with 10mM Ca^2+^ in pH 5 to induce MUC5B condensation (Fig. 2A) (8). We observed mostly linearized polymer chains under vehicle conditions, and condensed polymers that formed highly overlapping networks under high Ca^2+^/ low pH (Fig. 2A). MUC5B treated with sialidase primarily presented as highly entangled molecules, where the polymers frequently overlapped with themselves and did not take on a fully extended form, indicative of less electrostatic stiffening of the mucin (Fig. 2A). A minimum of 72 polymers per condition were categorized into either linear, entangled, or condensed morphologies based on a previously established scoring method (Fig. 2B) (6, 8). Sialidase treated MUC5B was enriched with highly entangled molecules (43.0% linear, 44.4% entangled, 12.5% condensed; P < 0.0001) compared to vehicle treated MUC5B, which primarily exhibited linearized polymer chains (93.1% linear, 6.8% entangled, 0.0% condensed). MUC5B exposed to high Ca^2+^/low pH exhibited substantially more condensed polymers s (34.1% linear, 31.8% entangled, 34.1% condensed; P < 0.0001) (Fig. 2A–B). These data suggest that reduced sialylation contributes to compaction of secreted MUC5B.

To further define the relationship between mucin conformation and sialylation, we performed rate zonal centrifugation on purified salivary MUC5B using a linear 10–35% sucrose gradient to separate mucin by size and shape. Mucins that sediment faster during rate zonal centrifugation have been described as more compact and pathologic, while mucins that sediment slower exhibit an expanded (linear) conformation (6, 9). We therefore hypothesized that slower sedimenting MUC5B would have a higher degree of sialylation, while faster sedimentation would be associated with reduced sialylation/charge. After rate zonal centrifugation, we collected fractions from the top of the gradient and subjected them to slot blotting for MUC5B and sialic acid. While MUC5B sedimented over fractions 3 to 11, the majority of sialylated MUC5B glycoforms were observed in the less dense (slower) sedimenting fractions (3 through 5) (Fig. 2C–D). TEM images of pooled fractions from different sedimentation rates across the gradient show that the slowest sedimenting, highest sialylated MUC5B (Fractions 3–5) represent primarily linearized polymers, while faster sedimenting, less sialylated MUC5B (Fractions 11–14) represent more condensed and less expanded polymers (Fig. 2E). Overall, these data indicate that mucin sialylation plays a major role in mucin linearization.

### Sialyltransferase Inhibition Impairs Mucociliary Transport in Primary HBECs

**Figure 3:**
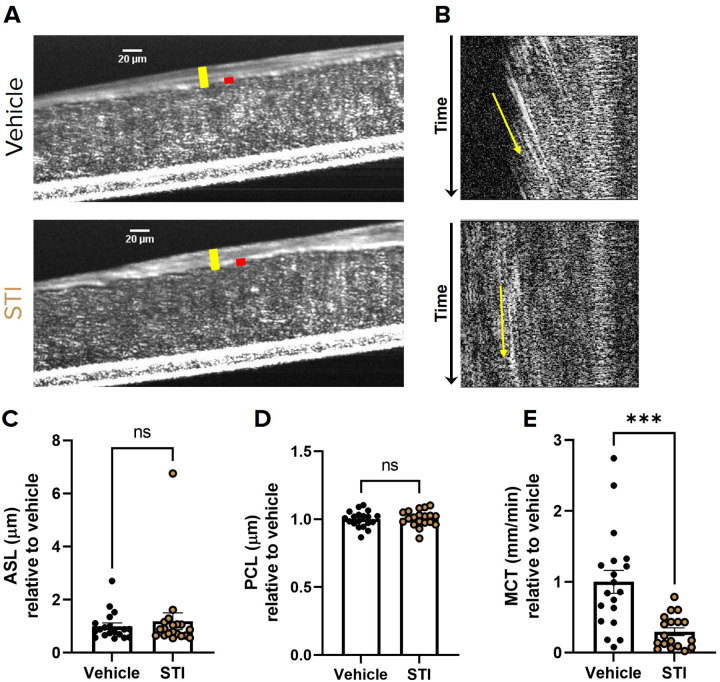
Sialyltransferase inhibition impairs mucociliary transport in primary HBECs **(A)** Representative μOCT images of HBECs treated with either vehicle or 200 μM STI demonstrate no differences in ASL (yellow bar) or PCL (red bar). **(B)** Reprocessed M-mode (e.g., x vs time) μOCT images show tracks of mucus particles above the epithelial surface of HBECs treated with vehicle or 200 μM STI; the more horizontal direction of particle streaks (yellow arrow) indicates more rapid transport. Summary data shows the effect of STI on **(C)** ASL and **(D)** PCL depths and **(E)**MCT. Regions of interest were measured and averaged for each HBEC filter. N=18–19/condition, representing 3 donors. Measurements were normalized to vehicle for each donor. nsP>0.05, ***P<0.001 by unpaired T-test or Mann-Whitney. Scale bars, 20μm.

To determine the consequences of reduced mucin sialylation on mucus transport, we treated non-CF HBECs with the sialyltransferase inhibitor 3Fax-Peracetyl Neu5Ac (STI) or DMSO vehicle for 24 hours and subsequently imaged them via μOCT (13, 30, 31). To ensure we studied only mucus biosynthesized under sialyltransferase inhibited conditions, we stimulated release of pre-existing mucin granules by purinergic stimulation with UTP prior to sialyltransferase treatment (32). HBECs treated with STI had significantly slower MCT rates (0.29 ± 0.05 mm/min; P < 0.001; Fig. 3B&E, S1 and S2 Videos) compared to vehicle treated cells (1.0 ± 0.16 mm/min). In contrast to effects on MCT rate, treatment with STI had no effect of the hydration state of the mucus layer as indicated by ASL and PCL depths (Fig. 3A–D) or CBF (SI Fig. 1A). Together, these data provides preliminary evidence that adequate sialylation of secreted mucin is vital for normal mucus transport, and this phenomenon occurs independently from the hydration state of the mucus.

### Sialyltransferase Inhibition Impairs Mucociliary Transport in Rat Tracheae

**Figure 4:**
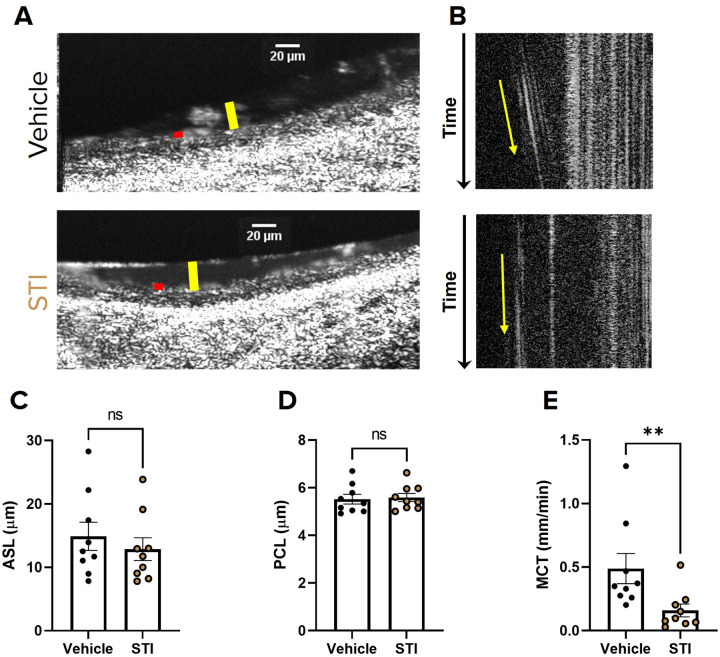
Sialyltransferase inhibition impairs mucociliary transport in rat tracheae **(A)** Representative μOCT images of excised WT rat tracheae from rats, treated with either PBS vehicle or 500 μM STI by intratracheal instillation daily for 7 days, demonstrate no differences in ASL (yellow bar) or PCL (red bar). **(B)** Reprocessed M-mode (e.g., x vs time) μOCT images show tracks of mucus particles above the epithelial surface of rat tracheae treated with vehicle or 500 μM STI; the more horizontal direction of particle streaks (yellow arrow) indicates more rapid transport. Summary data shows the effect of STI on **(C)** ASL and **(D)**PCL depths and **(E)** MCT. Regions of interest were measured and averaged for each trachea. N=9/condition. nsP>0.05, **P<0.01 by unpaired T-test or Mann-Whitney. Scale bars, 20μm.

To better understand the consequences of inhibiting sialylation of mucins *in vivo* and in the context of mucin-rich gland secretions, we administered STI (500μM) to WT rat trachea by intratracheal instillation daily for 7 days. (33, 34). The day before the last treatment, tracheae were excised and imaged via μOCT to evaluate the airway microanatomy. Consistent with the effects of STI on HBECs, tracheae treated with STI had significantly slower MCT (0.16 mm/min ± 0.05; P < 0.01; Fig. 4B,E, S3 and S4 Videos) compared to vehicle treated tracheae (0.49 ± 0.12 mm/min). Additionally, there was no difference in ASL or PCL depth (Fig. 4A,C–D), indicating that STI had no effect on airway hydration similar to our findings in HBE cells. CBF was also unaffected (SI Fig. 1B). These data support that reduced sialylation impairs mucus transport independent from hydration in an *in vivo* mucus model and in the presence of mucus glands.

### α−2,3 Sialyltransferase Inhibition Alone is Sufficient to Impair Mucociliary Transport

**Figure 5:**
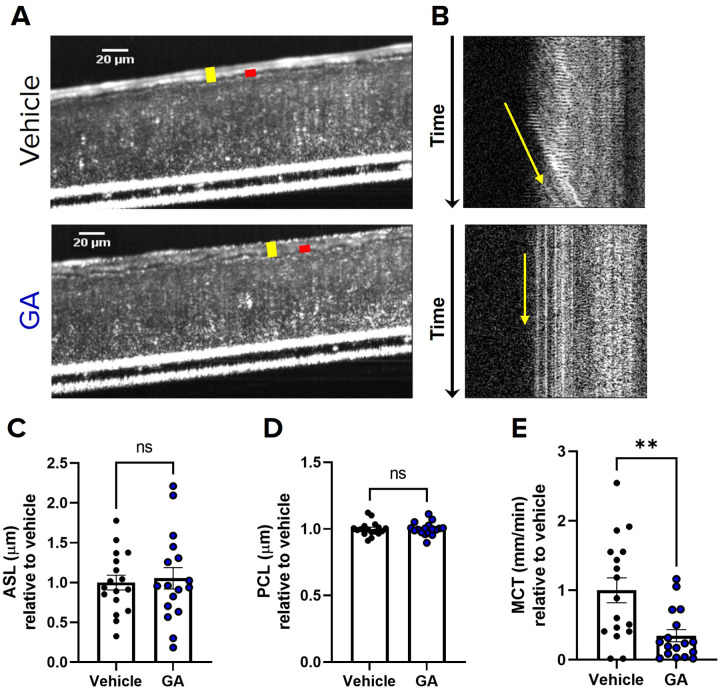
α−2,3 sialyltransferase inhibition alone is sufficient to impair mucociliary transport in HBECs **(A)** Representative μOCT images of HBECs treated with either vehicle or 120 μM GA demonstrate no differences in ASL (yellow bar) or PCL (red bar). **(B)** Reprocessed M-mode (e.g., x vs time) μOCT images show tracks of mucus particles above the epithelial surface of HBECs treated with vehicle or 120 μM GA; the more horizontal direction of particle streaks (yellow arrow) indicates more rapid transport. Summary data shows the effect of GA on **(C)** ASL and **(D)** PCL depths and **(E)**MCT. Regions of interest were measured and averaged for each HBEC filter. N=17/condition, representing 3 donors. Measurements were normalized to vehicle for each donor. nsP>0.05, **P<0.01 by unpaired T-test or Mann-Whitney. Scale bars, 20μm.

**Figure 6:**
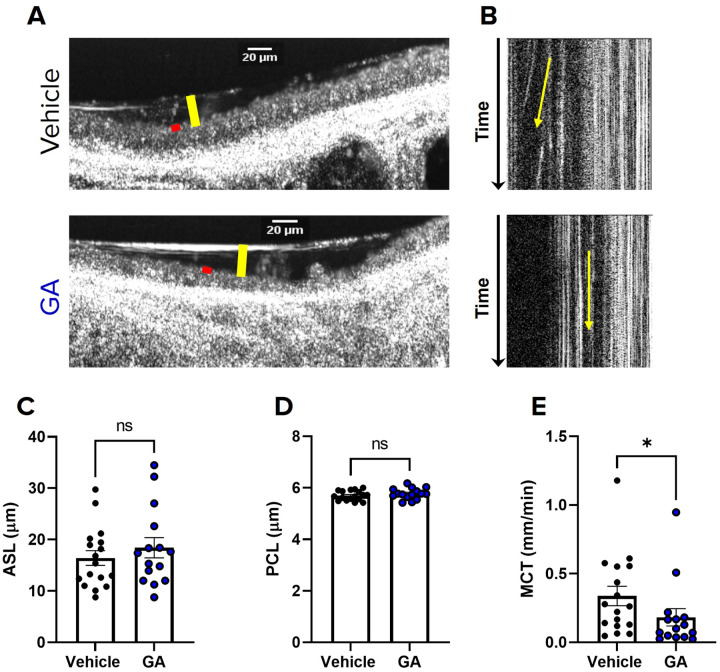
α−2,3 sialyltransferase inhibition alone is sufficient to impair mucociliary transport in trachea **(A)** Representative μOCT images of excised WT rat tracheae from rats, treated with either PBS vehicle or 300 μM GA by intratracheal instillation daily for 7 days, demonstrate no differences in ASL (yellow bar) or PCL (red bar). **(B)** Reprocessed M-mode (e.g., x vs time) μOCT images show tracks of mucus particles above the epithelial surface of rat tracheae treated with vehicle or 300 μM GA; the more horizontal direction of particle streaks (yellow arrow) indicates more rapid transport. Summary data shows the effect of GA on **(C)** ASL and **(D)** PCL depths and **(E)**MCT. Regions of interest were measured and averaged for each trachea. N=15–17/condition. nsP>0.05, *P<0.05 by unpaired T-test or Mann-Whitney. Scale bars, 20μm.

Since mucins contain mostly α−2,3 linked sialylated O-glycans (35–37) and evidence suggests that α−2,3 linked sialylation is selectively expressed in mucus producing goblet cells (38) we hypothesized that inhibition of α−2,3 sialylation alone would be sufficient to impair mucus transport. To determine the consequences of on mucus transport, we treated non-CF HBECs were treated with 120μM GA, an α−2,3 specific sialylation inhibitor, or vehicle for 24 hours and assessed for mucus physiology via μOCT (13, 39, 40). Similar to our findings with STI, cells treated with GA had significantly impaired MCT (0.35 ± 0.09 mm/min; P < 0.01; Fig. 5B,E, S5 and S6 Videos) compared to vehicle treated cells (1.0 ± 0.18 mm/min), and GA had no effect on ASL or PCL (Fig. 5A,C–D) or CBF (SI Fig. 1C). Consistent with this, rat tracheae instilled with 300μM GA in WT daily for 7 days exhibited a significant impairment in MCT (0.18 ± 0.06 mm/min; P < 0.05; Fig. 6B,E, S7 and S8 Videos) compared to those treated with vehicle (0.34 ± 0.07 mm/min). Furthermore, tracheae treated with GA showed no differences in ASL, PCL, or CBF (Fig. 6A,C–D; SI Fig. 1D). Altogether, these findings provide increasing evidence that normal sialylation, and specifically α−2,3 sialylation alone, may be vital for healthy mucus transport *in vitro* and *in vivo*, and that this mechanism is occurring independent from airway hydration.

### ST3Gal1 Expression is Decreased in CF and Increased with CFTR Modulation in HBECs

**Figure 7:**
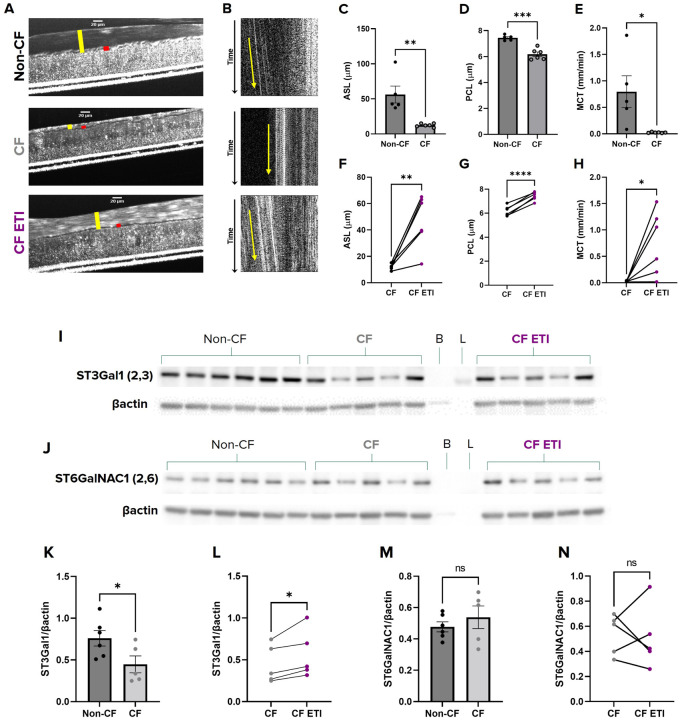
MCT and ST3Gal1 expression are decreased in CF and increased with CFTR modulation in HBECs **(A)** Representative μOCT images of non-CF HBECs treated with vehicle and CF HBECs treated with either vehicle or ETI triple modulators for 72hrs demonstrate decreased ASL (yellow bar) and PCL (red bar) in CF HBECs that were increased with ETI treatment. **(B)** Reprocessed M-mode (e.g., x vs time) μOCT images show tracks of mucus particles above the epithelial surface of non-CF HBECs treated with vehicle and CF HBECs treated with vehicle or ETI modulators; the more horizontal direction of particle streaks (yellow arrow) indicates more rapid transport. Summary data shows the differences in **(C)** ASL and **(D)** PCL depths and **(E)** MCT between non-CF and CF and (F-H) CF after ETI correction. Regions of interest from 4 different filters were measured and averaged for each HBEC donor. **(I)** Representative western blot of cell lysates probed for ST3Gal1 from non-CF HBECs treated with vehicle and CF HBECs treated with either vehicle or ETI triple modulators for 72hrs, demonstrate decreased expression of ST3Gal1 in CF HBECs that was partially restored after ETI treatment. **(J)**Representative western blot of cell lysates probed for ST6GalNAC1 from non-CF HBECs treated with vehicle and CF HBECs treated with either vehicle or ETI triple modulators for 72hrs, demonstrate no differences in expression of ST6GalNAC1. Quantification by densitometry of ST3Gal1 expression showing **(K)**a significant decrease in ST3Gal1 expression in CF HBECs compared to non-CF and **(L)** an increase in paired ETI treated CF HBECs. Quantification by densitometry of ST6GalNAC1 expression showing no differences in ST6GalNAC1 expression between **(M)** non-CF and CF or **(N)** paired ETI treated CF HBECs. N=5–6 donors/condition. nsP>0.05, *P<0.05, **P<0.01 by unpaired or paired T-test as appropriate. Scale bars, 20μm. Densitometry is presented normalized to βactin.

Changes in terminal mucin sialylation have been documented in CF, but the clinical implications of this remain poorly understood (23, 24, 41). To determine a potential impact of mucin sialylation in CF airway disease, we next evaluated sialyltransferase expression of the predominant ST3Gal and ST6GalNAC isoforms, ST3Gal1 and ST6GalNAC1, expressed in mucus secreting epithelial cells (42, 43).. We treated CF HBECs with the triple modulator combination, ETI, for 72hrs to restore CFTR function (44, 45). Non-CF cells and paired CF cells were each treated with vehicle control. Prior to sialyltransferase evaluation, we measured mucus physiology via μOCT. As expected, CF HBECs had significantly depleted ASL and PCL depths (ASL 12.4 ± 1.0 μm; P < 0.01; PCL 6.2 ± 0.2 μm; P < 0.001; Fig. 7A,C–D) compared to non-CF HBECs (ASL 56.2 ± 12.0 μm; PCL 7.4 ± 0.1 μm). CF HBECS also showed impaired MCT (0.02 ± 0.003 mm/min; P < 0.05; Fig. 7B,E, S9 and S10 Videos) compared to non-CF HBECs (0.80 ± 0.3 mm/min). Furthermore, ETI treatment of CF HBECs significantly restored ASL and PCL depths (ASL 46.9 ± 8.1 μm; P < 0.01; PCL 7.4 ± 0.14 μm; P < 0.0001; Fig. 7A,F–G), and improved MCT (0.75 ± 0.25 mm/min; P < 0.05; Fig. 7B,H, S11 Video). These data showed expected phenotypic differences in mucus transport in the presence and absence of CFTR function, allowing us to interrogate how this relates to the forms of MUC5B present.

Following μOCT evaluation, HBECs were collected and cell lysates immunoblotted for ST3Gal1 and ST6GalNAC1, the two primary siaylatransferases responsible for O-linked α−2,3 and α−2,6 sialylation, respectively (12, 46). ST3Gal1 expression was significantly lower in CF HBECs (0.45 ± 0.1 ST3Gal1/βactin; P < 0.05; Fig. 7I,K) than that of non-CF HBECs (0.76 ± 0.1 ST3Gal1/βactin). CF HBECs treated with ETI showed significantly increased expression of ST3Gal1 (0.56 ± 0.13 ST3Gal1/βactin; P < 0.05;Fig. 7I,L) when compared to their paired, vehicle treated CF HBECs. There were no notable differences in ST6GalNAC1 expression when comparing non-CF, CF, or CF HBECs post ETI treatment (Fig. 7J,M–N). In summary, these data provide evidence that sialylation is dysregulated in CF muco-obstructive disease, and that this finding is linked to CFTR function.

## Discussion

In muco-obstructive diseases, such as CF, mucus stasis has largely been attributed to airway dehydration and mucus hyper-concentration (3, 47), but recent evidence shows that electrostatic abnormalities of gel-forming mucins, MUC5B and MUC5AC, also contribute to aberrant mucus physiology (8, 9, 48). Although sialic acid highly contributes to mucin charge and electrostatics, the exact role of sialylation on the physiological and biophysical properties of mucin remains vastly understudied. Here, for the first time, we demonstrate the consequences of reduced mucin sialylation on the biophysical properties of MUC5B and the functional consequences of sialylation inhibition on mucus transport *in vitro* and *in vivo*. Furthermore, we identify decreased expression of ST3Gal1 in CF HBECs. Overall, our findings indicate that aberrant MUC5B sialyation occurs in CF lung disease, resulting in compact mucin forms that contribute to abnormally delayed mucociliary transport.

The glycosylation profiles of mucins can be heterogeneous, resulting in multiple glycoforms. Previous reports evaluating the charge states of MUC5B and MUC5AC demonstrated that MUC5AC exists as a single major charge form, while MUC5B exists in two major charge forms, denoted as “high” and “low” charge forms (19). The high charged form predominates in healthy airway secretions, while the low charged form is more abundant in several muco-obstructive diseases including CF, COPD, and asthma. Furthermore, studies have demonstrated increased levels of the low charged MUC5B form within viscous mucus plugs (20–22, 27). This suggests that decreased mucin charge may bear pathological significance in muco-obstructive diseases through increased mucus compaction and transport impairment. Here, using the same technique that initially identified the two charge variants of MUC5B, we show that the highest charged species of mucin also has the strongest sialic acid detection by WGA lectin blotting of MUC5B from cell secretions (Fig. 1 A). Furthermore, we show that sialylation reduction decreased the charge state of MUC5B and produced a low charged MUC5B similar to that observed in pathologic mucus (Fig. 1B). Although other modifications, such as sulfation, are likely to also play a role in determining the charge state of MUC5B, reducing sialylation alone was sufficient to obtain the lower MUC5B charge form that is consistent with previously published reports in other muco-obstructive diseases (20, 21, 27).

High negative charge density on mucins has been shown to promote stiffening of the mucin backbone through repulsion of neighboring charges, which is an important feature in the maturation of mucin polymers and the formation of the mucus gel (10, 49, 50). During packaging and prior to secretion, these anionic charges are stabilized by divalent Ca^2+^, allowing the mucin to condense for packaging and transport (6, 18, 51). Upon secretion into the airway, Ca^2+^ is chelated by bicarbonate, freeing these charges to repel and extend the mucin backbone (7, 16, 52). Therefore, loss of these charges would be expected to weaken these repulsive forces and hinder mucin expansion after secretion. In support of this, we show a significantly increased occurrence of entangled polymers after sialidase treatment of salivary MUC5B (Fig. 2A–B), suggesting that decreased MUC5B sialylation increases mucin compaction. Interestingly, the conformation of sialidase treated MUC5B, resembles the morphology of the previously reported low charged MUC5B from a mucus plug, which was also composed of mostly entangled and non-linear polymers (similar to our observations) (22). The low charge or reduced sialylated form of MUC5B may contribute to more compaction of the mucin and impaired expansion. Evidence for this was also demonstrated through rate zonal centrifugation, where the degree of sialylation strongly correlated with the sedimentation of MUC5B (Fig. 2). Here, we show that the slower sedimenting MUC5B forms that are mostly linear and mature contain higher amounts of sialic acid. Several studies are congruent with this finding and show slower sedimenting MUC5B during rate zonal centrifugation exhibits a more expanded conformation that is more fully extended and mature (6, 8). Overall, these data provide evidence that higher levels of mucin sialylation facilitate linearization of secreted mucin, a feature imperative for clearance of mucus.

We utilized sialyltransferase inhibitors to reduce the sialylation of secreted mucins *de novo*, which allowed us to evaluate the functional consequences of reduced mucin sialylation on MCT, where both MUC5B and MUC5AC are present and contribute to mucus clearance (13, 30, 40, 53). We show that decreased sialylation of secreted mucin by STI significantly impaired MCT and had no effects on ASL, PCL, or CBF in both non-CF HBECs and WT rat tracheae (Fig. 3–4). In particular, inhibition of 2,3 sialyltransferase alone was sufficient to recapitulate this phenotype of impaired MCT without affecting hydration (Fig. 5–6). In addition to secreted mucins, ciliated epithelia are lined with membrane bound (tethered) mucins that are important for PCL hydration and ciliary beating (54, 55). It is possible that reduced sialylation of tethered mucins may have contributed to the impaired MCT observed; however, it is more likely that the impaired MCT was due to reduced sialylation of secreted mucins, since we observed no changes in ASL or PCL depth or CBF. Additionally, previous work shows that ciliated epithelium primarily express α−2,6 linked sialylation while mucin secreting goblet cells selectively express α−2,3 linked (38). Our findings are likely consequence to reduced sialyation of secreted mucin, since we observed a decrease in MCT after α−2,3 specific inhibition. These findings not only underscore the importance of normal sialylation for mucociliary clearance but also show that the relationship between mucin sialylation state and MCT is most likely attributed to the abundance of 2,3 sialylation on O-linked glycans (37–39).

CFTR has been suggested to regulate terminal glycosylation of mucins (23, 24, 41), but whether this is due to CFTR dependent anion transport is unknown. Some studies document changes in mucin sialylation as a result of infection in CF (56, 57), while others have reported altered mucin sialylation in CFTR^−/−^ newborn piglets before the onset of inflammation or infection (58). Interestingly, there is also evidence that sialylation may be altered as a direct consequence of the defective CFTR due to its role in organelle acidification or protein turnover both of which could affect glycosyltransferases including the sialyltransferases (25, 59, 60). Here, we measured sialyltransferase protein expression in non-CF, CF, and CF-ETI corrected HBECs. We found that ST3Gal1 protein was significantly lower in CF HBECs compared to non-CF (Fig. 7). Additionally, ST3Gal1 expression was significantly increased after 72hr ETI treatment suggesting that CFTR correction may augment ST3GAL1 expression. Future studies are required to determine the relationship between CFTR and ST3Gal1 expression. Nevertheless, our findings suggest that dysregulated sialylation of mucin may be a contributing factor and therapeutic target in CF muco-obstructive disease.

**Figure 8:**
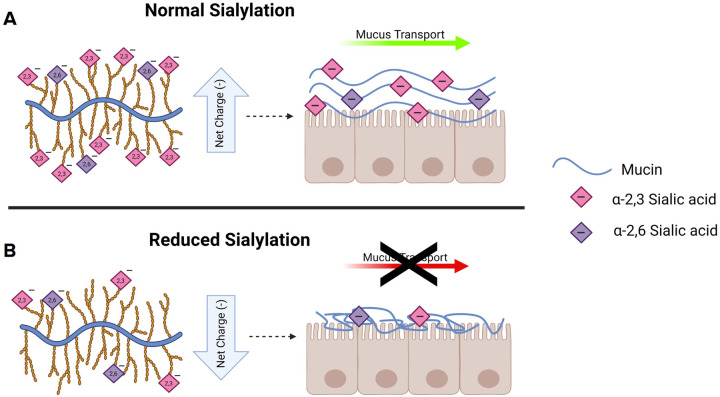
The consequences of reduced sialylation on mucus physiology **(A)** Under normal conditions, gel-forming mucins are heavily sialylated on the terminus of their glycan chains. This sialylation produces a high negative charge density across the mucin backbone. These negative charges promote mucin expansion and linearization through charge repulsion of neighboring sialic acids within and across mucin polymers. This promotes the formation of a normal mucin network and facilitates mucus transport. **(B)** When the level of sialylation is reduced, the net charge across the mucin backbone is also reduced. The charge deficit ameliorates the repulsive forces within and between mucin polymers and results in less linear, more entangled mucins. This results in an aberrant mucin network and ultimately impairs mucus transport.

In summary, we demonstrate the consequences of reduced mucin sialylation on mucin charge state, mucin confirmation, and mucus transport. Our data supports a novel model in which sialylation promotes normal MUC5B linearization and MCC by increasing mucin charge state (Fig. 8A). Conversely, when sialylation/charge is reduced as observed in CF HBECs, MUC5B becomes entangled and MCC is impaired (Fig. 8B). In addition, we posit that reduced expression of ST3GAL1 in CF, which can be corrected by ETI, may contribute to reduced mucin charge, expansion, and mucus clearance. Furthermore, these findings provide impetus for evaluating mucin sialylation and cognate transferases as therapeutic targets to combat mucus stasis in a plethora of muco-obstructive diseases.

## Methods

### Sex as a Biological Variable

Our study examined male and female animals, and similar findings are reported for both sexes.

### Primary HBE Cell Culturing

Primary human bronchial epithelial cells (HBECs) harvested from lung explants of previously healthy (Non-CF) or F508del-CFTR homozygous (CF) donors. First or second-passage cells, were seeded onto 6.5-mm-diameter permeable supports (Corning Inc., Corning, NY) coated with NIH 3T3 fibroblast conditioned media at a density of 0.5 × 10^6^ cells per filter. Cells were grown in in PneumaCult^™^-ALI Medium (STEMCELL Technologies, Canada) to induce terminal differentiation at air liquid interface for at least 4 weeks (61, 62). Prior to all studies, primary HBECs were treated apically with 100uM UTP in PBS for 40 minutes to induce granular mucin secretion to remove pre-existing intracellular mucin produced before experimental conditions (32).

### Rat Model

All animal experiments at UAB were conducted in accordance with UAB Institutional Animal Care and Use Committee (IACUC) approved protocols. All animal experiments used wild-type (WT) Sprague-Dawley rats. Animals were bred and housed in standard cages with a 12-h light/dark cycle with ad libitum access to food and water and were routinely monitored. Rats of the same sex were co-housed from time of weaning to study conclusion. Weaned rats were maintained on a standard rodent diet. Animals were euthanized by intraperitoneal injection of 500μL pentobarbital sodium (390 mg/mL) followed by exsanguination of the hepatic portal vein. Animals used in this study were ≥ 6 months to allow maturation of submucosal glands (33). Male and Female rats were used and all experimental groups were matched by age and sex.

### Sialyltransferase Inhibition

Terminal differentiated HBECs at ALI were treated with 200μM 3Fax-Peracetyl Neu5Ac (STI) to block sialylation, or 120μM glycolithocholic acid (GA) to specifically inhibit α−2,3 sialylation, in the basolateral compartment for 24 hours and then imaged via μOCT (13). Control cells were treated with DMSO vehicle. To inhibit sialylation in rat tracheae, 100μL of 500μM STI or 300μM GA diluted in PBS, was intratracheally instilled in WT rats daily for seven days following established methods (63). Control groups were treated with DMSO vehicle diluted in PBS.

### μOCT Imaging and Analysis

Measurements of the functional microanatomy of primary HBECs or freshly excised rat tracheae were performed using micro-optical coherence tomography (μOCT), a high-speed, high-resolution microscopic imaging modality as previously described (64). The μOCT instrument provides cross-sectional images of the epithelium with a sub-cellular resolution sufficient to directly visualize and quantify micro-anatomic parameters including air surface liquid (ASL) depth, periciliary liquid (PCL) depth, mucociliary transport (MCT) rates, and ciliary beat frequency (CBF). Images were acquired at a rate of 40 frames per second and at 512 A-lines per frame. ASL and PCL depths were quantified by direct geometric measurement of the respective layers with a correction factor based the estimated refractive index of n = 1.33, using ImageJ (NIH) software (62). MCT rate was determined using time elapsed and distance traveled of native particles in the mucus layer over multiple frames. Ciliary beat frequency (CBF) and was investigated by Fourier analysis of the time-varying reflectance due to beating cilia using MATLAB. For consistency, HBECs were measured at four standardized locations for each culture. Tracheae were placed in the same proximal to distal orientation and the imaging beam was placed at six standardized locations along the ventral surface of the trachea as previously described (33, 62).

### Native MUC5B Isolation

Whole human saliva from a healthy donor was collected by chewing on Parafilm to stimulate secretion and collected into a 50-ml falcon tube. Saliva was centrifuged at 3000 x g for 25 minutes at 4°C to remove cells and debris. Clarified saliva was solubilized overnight in 0.1M NaCl 20mM/Tris pH 7.4 at 4°C with rotation. Following solubilization, cesium chloride (CsCl) was added to a starting density of 1.45g/mL, and saliva was fractionated by isopycnic density centrifugation in a Beckman SW41 Ti swinging bucket rotor at 118k x g for 72hrs at 15C (8, 29). Following centrifugation, MUC5B containing fractions were pooled and buffer exchanged into 10mM NaCl/10mM Tris, pH 7.4.

### MUC5B Treatments

To evaluate the role of sialylation on charge state of MUC5B, secreted mucus from non-CF HBECs was collected and solubilized in 4M guanidinium hydrochloride (GuHCl). Following solubilization, mucins were partially purified and subjected to a neuraminidase buffer exchange containing 50mM sodium acetate, 4mM CaCl_2_ pH 6 using a 100kDa Amicon Ultra centrifugation column (Millipore Sigma, Burlington, MA). Partially purified mucin was split into equal (5μg) aliquots and treated with increasing amounts of neuraminidase from *Vibrio Cholerae* (Roche, Bavaria, Germany), ranging from 0mU/mL to 25mU/mL, for 2hrs at 37°C. Neuraminidase vehicle was added to equalize volumes. After incubation, a denaturing buffer containing 6M urea and 25mM dithioreitol (DTT) was added to inactivate sialidase before electrophoresis.

To evaluate the consequences of reduced sialylation on MUC5B conformation via TEM, 50μg/mL of MUC5B purified by CsCl gradient centrifugation was treated with either neuraminidase vehicle or 12.5mU/mL neuraminidase for 2hrs at 37°C. CaCl_2_ was added to a final of 1mM to enable neuraminidase activity. After treatment, 10mM EGTA, pH7.4 was added to vehicle and neuraminidase treated mucin to remove CaCl_2_. As a positive control, 10mM CaCl_2_ was added to vehicle treated mucin and adjusted to pH 5. Samples were incubated overnight at 4°C prior to negative staining.

### Agarose-polyacrylamide Gel Electrophoresis

Following partial purification and neuraminidase treatment, secreted mucus from non-CF HBECs was buffer exchanged into gel-loading buffer containing 6M Urea, 25mM DTT, and 0.1% SDS and heated at 95°C for 10 minutes. Mucins were separated by their inherent charge on an agarose-polyacrylamide-urea gel as previously described (65). Briefly, gels were prepared with 1% agarose, 1.5% polyacrylamide, and 4M urea. Sample (5μg) was loaded and electrophoresed at 80V for 2hrs, followed by transfer at 40V for 6 hours onto a 0.45μm nitrocellulose membrane. Separate, identical blots were incubated with either MUC5B primary antibody (4A10-H2, Novus Biologicals, Centennial, CO) at 1:500 dilution followed by mouse anti-IgG horse radish peroxidase (HRP) conjugated secondary (31340, Invitrogen, Waltham, MA) at 1:3000 dilution or 1μg/mL biotinylated WGA (B-1025–5, Vector Labs, Newark, CA) followed by Vectastain ABC-HRP reagent at 1:2000 dilution. HRP activity was detected using enhanced chemiluminescence detection solution (Bio-Rad, Hercules, CA) and imaged on a Bio-Rad Gel Doc XR Gel Documentation System.

### Transmission Electron Microscopy and Evaluation

Samples were adjusted to 5μg/mL and incubated for 30 seconds on carbon coated CF400-Cu grids (EMS, Hatfield, PA) that had been glow discharged at 30 Volts for 30 seconds. Grids were washed in ddH2O for 10 seconds and then negative stained with 2% (w/v) uranyl acetate for 1 minute (8). TEM data were recorded using a JEOL JEM-1400Flash microscope (JEOL USA, Peabody, MA) at 120 Kv in a magnification range between 30,000 to 50,000x. MUC5B polymers from vehicle, sialidase, and 10mM calcium pH 5 conditions were counted and categorized, based on their appearance, into three groups: condensed, entangled, or linearized (8). A total of 30–35 images were collected for each condition, representing 72–85 polymers per condition. Images were blinded prior to polymer scoring.

### Rate Zonal Centrifugation

Rate zonal centrifugation was performed on 11mL linear 10–35% sucrose gradients in PBS, pH 7.4. Gradients were prepared as 4-step discontinuous gradients and thawed at 4°C for 20 hours prior to centrifugation to form linear gradients (66). 500μl of sample was layered onto the top of the gradient and centrifuged in a SW41 Ti swinging bucket rotor at 210k x g for 90 minutes at 15°C (8). Following centrifugation, the gradient was fractioned from the top in 500μL increments, giving 23 total fractions. The fractions were evaluated for mucin and sialic acid content.

### Slot Blotting and Detection

Equal volumes of samples were diluted in buffer containing 4M GuHCl, 25mM DTT, 0.1M Tris pH 7.4 and heated at 95°C prior to loading. Samples (200uL) were transferred onto a 0.45μm nitrocellulose membrane under gentle vacuum using Bio-Rad Bio-Dot SF microfiltration apparatus (Bio-Rad, Life Sciences, USA). MUC5B content was evaluated using anti-human MUC5B primary antibody (4A10-H2, Novus Biologicals, Centennial, CO) at 1:500 dilution followed by IRDye^®^ 680RD Goat anti-Mouse IgG Secondary Antibody (Li-COR, Lincoln, NE) at a 1:10,000 dilution. Sialic acid content was evaluated using SIAFIND^™^ biotinylated Pan-Specific Lectenz (Lectenz Bio, Athens, GA) at 20μg/mL followed by IRDye^®^ 800CW Streptavidin (Li-COR) at a 1:5000 dilution. Secondary antibodies were visualized, and densitometry performed on band intensities using a LI-COR Odyssey^®^ CLx Infrared Imaging System (Li-COR Biosciences, Lincoln, USA).

### Elexacaftor tezacaftor ivacaftor (ETI) Treatment on CF HBECs

Well differentiated CF HBECs, homozygous for F508del-CFTR, at ALI were treated with the three drug cystic fibrosis transmembrane conductance regulator (CFTR) modulator combination of 3μM Elexacaftor (E, VX-445), 3μM Tezacaftor (T, VX-661), and 1μM Ivacaftor (I, VX-770, Selleck Chemicals LLC, Houston, TX), or DMSO vehicle in the basolateral compartment for 72hrs. Drugs were refreshed every 24hrs. Non-CF HBECs were treated with DMSO vehicle. After 72 hours, cells were imaged via μOCT and subsequently collected for sialyltransferase protein evaluation.

### Sialyltransferase Western Blotting

Following treatments, HBECs were lysed in RIPA buffer (RPI Corp, Mt. Prospect, Illinois) with Halt protease and phosphatase inhibitor cocktail (Thermo Scientific, Waltham, MA). Three filters per donor/condition were pooled and total protein concentration was determined by Bradford protein assay (Thermo Scientific, Waltham, MA). 15μg of protein per condition were loaded and separated by SDS-PAGE. Separate, identical blots were incubated with ST3Gal1 (PA5–21721, Invitrogen) or ST6GalNAc1 (PA5–31200, Invitrogen) primary antibody at 1:1000 dilution followed by rabbit anti-IgG horseradish peroxidase conjugated secondary antibody (31466, Invitrogen). HRP activity was detected using enhanced chemiluminescence detection solution (Bio-Rad, Hercules, CA) and imaged on a Bio-Rad Gel Doc XR Gel Documentation System for quantification. ImageJ (NIH) software was used to perform densitometry measurements (67).

### Statistics

Statistical analysis was performed in GraphPad Prism version 9 or greater. Data were tested for normality using Shapiro-Wilk’s test followed by non-parametric or parametric analysis when appropriate. All μOCT data comparing two groups (Figs. 3–6) and immunoblotting data were subjected to either a two-tailed, unpaired T-test when parametric or Mann-Whitney test comparing mean ranks when non-parametric. μOCT data comparing three groups were analyzed by Kruskal-Wallis test with Dunn’s post hoc to compare groups. The categorical scoring of TEM imaged mucin polymers was analyzed by Chi-Square to test occurrence. A p-value of less than 0.05 was considered statistically significant. Statistics are presented as mean ± SEM.

### Study Approval

Use of human bronchial epithelial cells was approved by the University of Alabama at Birmingham (UAB) Institutional Review Board (IRB) 300001383. Use of human saliva was approved by the UAB IRB 120523006. Written informed consent was received from all participants who provided sputum samples and for acquisition of airway tissues to procure primary human airway cells. All experiments were performed in accordance with relevant guidelines and regulations. Use of WT rats was approved by the UAB Institutional Animal Care and Use Committee (IACUC) IACUC-21806. All animal studies were reported in accordance with ARRIVE guidelines.

## Data Availability

Data is provided within the manuscript or supplementary information files.
